# Optimal effect-site concentration of remifentanil for preventing cough during removal of the double-lumen endotracheal tube from sevoflurane-remifentanil anesthesia: A prospective clinical trial

**DOI:** 10.1097/MD.0000000000003878

**Published:** 2016-06-17

**Authors:** Sook Young Lee, Ji Young Yoo, Jong Yeop Kim, Dae Hee Kim, Jung Dong Lee, Go Un Rho, Hyungbae Park, Sung Yong Park

**Affiliations:** a Department of Anesthesiology and Pain Medicine; b Office of Biostatistics, Ajou University, School of Medicine, Suwon, Korea.

**Keywords:** anesthesia, complications, extubation trachea, pharmacology, remifentanil

## Abstract

Opioids are used as a treatment for coughing. Recent studies have reported an antitussive effect of remifentanil during recovery from general anesthesia by suppressed coughing. The coughing reflex may differ throughout the respiratory tract from the larynx to the bronchi. But the proper dose of remifentanil to prevent cough during double-lumen tube (DLT) extubation is unknown.

Twenty-five ASA physical status 1 and 2 patients, 20 to 65 years of age who were undergoing video-assisted thoracoscopic lung surgery requiring 1-lung ventilation were enrolled. The effective effect-site concentration (Ce) of remifentanil for 50% and 95% of patients (EC_50_ and EC_95_) for preventing cough was determined using the isotonic regression method with a bootstrapping approach, following the Dixon up-and-down method. Recovery profiles and hemodynamic values after anesthesia were compared between patients with cough and patients without cough.

EC_50_ and EC_95_ of remifentanil was 1.670 ng/mL [95% confidence interval (95% CI) 1.393–1.806] and 2.275 ng/mL (95% CI 1.950–2.263), respectively. There were no differences in recovery profiles and hemodynamic values after anesthesia between patients with/without cough. No patients suffered respiratory complications during the emergence period.

Remifentanil can be a safe and reliable method of cough prevention during emergence from sevoflurane anesthesia after thoracic surgery requiring DLT. EC_50_ and EC_95_ of remifentanil that suppresses coughing is 1.670 and 2.275 ng/mL, respectively.

## 1 Introduction

Coughing during emergence from anesthesia and tracheal extubation can be associated with various adverse events such as laryngospasm, hypertension, tachycardia, and increased intracranial and intraabdominal pressure.^[1]^ Various methods have been tried to prevent coughing during emergence from anesthesia and tracheal extubation.^[1–4]^ Opioids are used as a treatment for coughing and recent studies have reported an antitussive effect of remifentanil during recovery from general anesthesia by suppressed coughing.^[5–7]^ The effective effect-site concentration (Ce) of remifentanil for 50% and 95% of patients (EC_50_ and EC_95_) that prevents cough during emergence after general anesthesia using single-lumen tube (SLT) has been determined.^[7–10]^

A double-lumen tube (DLT) is currently used to facilitate surgical exposure in patients undergoing intrathoracic surgical procedure. Use of the larger DLT increases the incidence of hoarseness and airway injury,^[11]^ and can stimulate different parts of the respiratory tracts compared with SLT. Some reports described that the coughing reflex differs throughout the respiratory tract from the larynx to the bronchi.^[12–17]^ But, to date, there is no report with regard to effective Ce of remifentanil for preventing cough during emergence using DLT. The proper dose of remifentanil needed to prevent coughing during DLT extubation.

The purpose of this study was to evaluate the EC_50_ and EC_95_ of remifentanil in effect-site concentration for preventing cough during emergence after general anesthesia using DLT.

## 2 Methods

The study was approved by the Institutional Review Board of Ajou University Hospital (Ref: AJIRB-MED-CT4-14-456). After written informed consent was obtained from participants, 25 American Society of Anesthesiologists physical status (ASA) physical status 1 and 2 patients, aged 20 to 65 years who were undergoing video-assisted thoracoscopic lung surgery requiring 1-lung ventilation in Ajou medical center were enrolled. Exclusion criteria were administration of an angiotensin-converting enzyme inhibitor, anticipated difficult airway, gastroesophageal reflux, asthma, chronic obstructive disease, and upper respiratory infection. The trial is registered in a public trial register (Clinical Research information Service, CRIS) under the identification number KCT0001563.

All patients were evaluated before surgery and were not premedicated. Patients were monitored with electrocardiography, arterial oxygen saturation (SpO_2_), noninvasive blood pressure, and bispectral index (BIS). For effect-site targeted-controlled infusion (TCI) of remifentanil, a commercial TCI pump (Orchestra Base Primea, Fresinus Vial, France) was used. The pumps were operated by the Minto pharmacokinetic model for remifentanil.^[18]^

All patients were preoxygenated with 100% oxygen for 1 minute and anesthesia was induced using intravenous propofol 1.5 mg/kg and effect-site TCI of remifentanil. Remifentanil infusion began at the time of anesthetic induction. After the patient did not response to verbal command, rocuronium 0.6 mg/kg was given intravenously and manual ventilation with 100% oxygen was done for 90 seconds. After confirming a BIS value below 60, tracheal intubation was performed in all patients with a 35-Fr DLT. The correct positioning of DLT was confirmed with fiberoptic bronchoscopy. If repositioning was necessary, the DLT was guided into position via bronchoscope. Cuff pressure was set to 20 to 25 mmH_2_0 with a hand pressure gauge. Anesthesia was maintained with sevoflurane and effect-site TCI of remifentanil at 2.5 to 4.0 ng/mL to maintain blood pressure and heart rate within 20% of baseline value, and to maintain a BIS target level of 40 to 60 during surgery. Lungs were ventilated with 50% oxygen during the initial 2-lung ventilation period and the fraction of oxygen was changed to 100% during the 1-lung ventilation period. End-tidal CO_2_ was maintained at 35 to 40 mm Hg during the operation. Patient's core temperature was maintained at 36.5 ± 0.5°C. After completing the surgery, effect-site concentration of remifentanil was titrated to a predetermined concentration (initial concentration being 2.0 ng/mL for the first patient). Immediately after the surgical procedure, sevoflurane was stopped and sugammadex 4 mg/kg was given for reversal of neuromuscular block. Intravenous ibuprofen 400 mg was given for pain control. The predetermined concentration was maintained at least 10 minutes throughout emergence for the effect-site concentration and plasma concentration were anticipated to be stable. The patient was verbally requested to open their eyes without any stimulus. When the patient did so, deep breathing was encouraged; after adequate tidal volume and ventilatory frequency were confirmed, the DLT was removed with cuff deflated.

Cough, defined as a sudden contraction of the abdominal muscle, was assessed during anesthetic emergence from discontinuation to 2 minutes after extubation. The predetermined concentration was decreased by 0.5 ng/mL for the next patient if the patient did not cough during emergence. Similarly, if the patient coughed anytime during emergence, smooth emergence was considered to have failed and the predetermined concentration was increased by 0.5 ng/mL. The anesthesiologist who was blinded to the remifentanil effect-site concentration performed the extubation, and checked patients for coughing. After extubation, patient was given 100% oxygen via a face mask for 5 minutes before transfer to the postanesthesia care unit after confirming stable vital signs.

Bradypnea, defined as a respiratory rate <8 rates per minute or a SpO_2_ <95% despite oxygen supplement and other respiratory complications were assessed during the emergence period. Mean arterial pressure and heart rate, SpO_2_, and BIS were recorded after completion of operation, immediately before and after extubation, and 2 minutes after extubation. Body temperature was recorded at the end of surgery. End-tidal CO_2_ and respiration rate were also recorded immediately after extubation. End-expiratory sevoflurane concentration at eye opening and the intervals from the discontinuation of sevoflurane to eye opening and to extubation were recorded.

For estimating EC_50_ and EC_95_ of remifentanil in preventing cough, the patients were enrolled until obtaining 6 crossover pairs according to the Dixon's sequential allocation method.^[7]^ Twenty-five patients were included to obtain stable estimations. The EC_50_ of remifentanil was determined by calcualting the average of the midpoint dose of all independent pairs of patients after 8 crossover points were obtained. The data were subjected to isotonic regression method for calculation of EC_50_ and EC_95_ with 95% confidence interval (CI).^[19]^ An adjusted response probability was calculated by the pooled adjacent-violators algorithm (PAVA), and the CI was estimated by a bootstrapping approach.^[20,21]^

All continous variables, except for estimated EC, are shown as the mean ± SD or median (interquartile range) and comparison between cough suppression patients and failed cough suppression patients for duration of suregry and anesthesia, end-tidal sevoflurane concentration at eye opening and extubation, time to eye opening, and extubation were made with a Wilcoxon rank sum test. The numbers of ASA physical status were compared using Fisher exact test. Hemodynamic data during emergence between cough suppression patients and failed cough suppression patients were analysed by a Wilcoxon rank sum test. R statistical software package version 3.3.3 (R Foundation for Statistical Computing, Vienna, Austria) was used for statistical analysis. *P* < 0.05 was considered statistically significant.

## 3 Results

Using the Dixon up-and-down methods, 25 patients were enrolled. Among them, 2 patients were excluded due to surgical factors (main bronchus injury and postoperative ventilator therapy). Finally, 23 patients completed all the assessments. The patients’ characteristics are presented in Table 1.

**Table 1:**
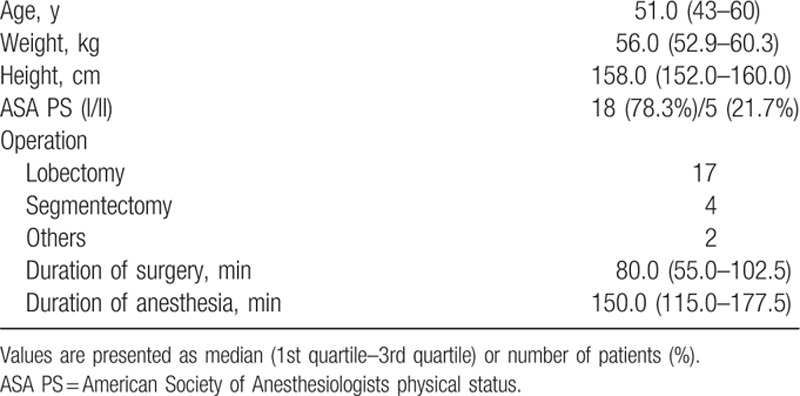
Demographic data.

**Figure 1:**
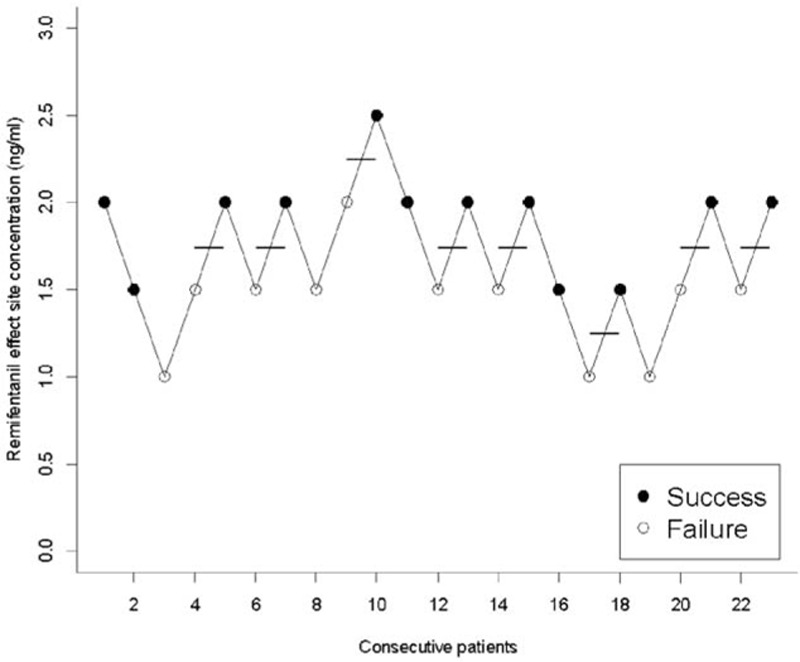
Assessment of success or failure to prevent cough during emergence with predertermined concentrations of remifentanil from consecutive patients by the Dixon up-and-down method.

**Figure 2:**
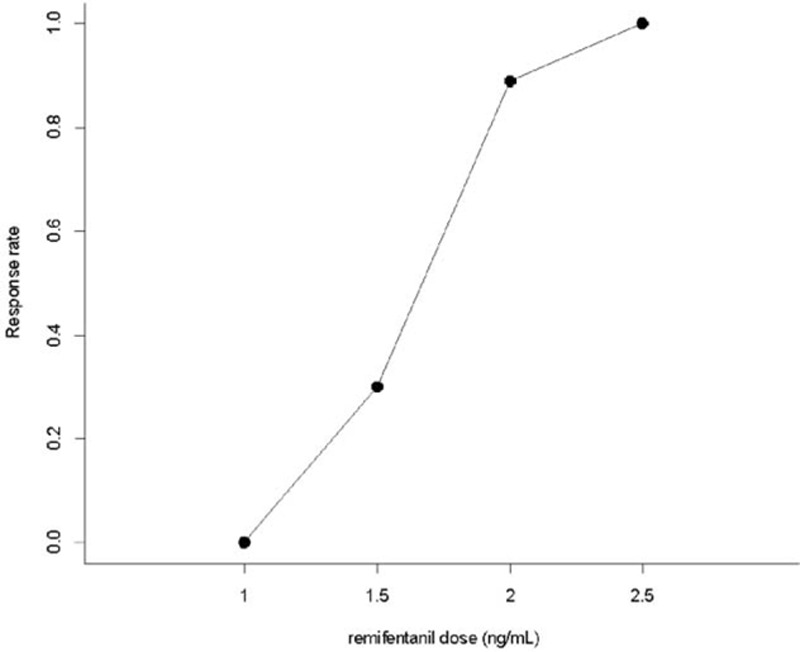
Pooled adjacent violators algorithm response rate. The EC_50_ of remifentanil was 1.670 ng/mL (95% CI 1.393–1.806). The EC_95_ of remifentanil was 2.275 ng/mL (95% CI 1.950–2.463). CI = confidence interval, EC_50_ = effective Ce of remifentanil for suppression of emergence cough in 50% of patients, EC_95_ = effective Ce of remifentanil for suppression of emergence cough in 95% of patients.

The up-and-down results in consecutive patients and PAVA response rate are shown in Figs. 1 and 2. The EC_50_ of remifentanil needed to prevent cough during emergence, estimated by the Dixon method, was 1.75 ng/mL ± 0.27. EC_50_ and EC_95_ of remifentanil as estimated by the isotonic regression model with a bootstrapping approach was 1.670 ng/mL (95% CI 1.393–1.806) and 2.275 ng/mL (95% CI 1.950–2.263), respectively.

The comparisons of recovery profiles and hemodynamic values after anesthesia between the patients without cough (n = 12) and those with cough (n = 11) are presented in Tables 2 and 3. There were no differences in recovery profiles and hemodynamic values after anesthesia between patients with cough and patients without cough except height.

**Table 2:**
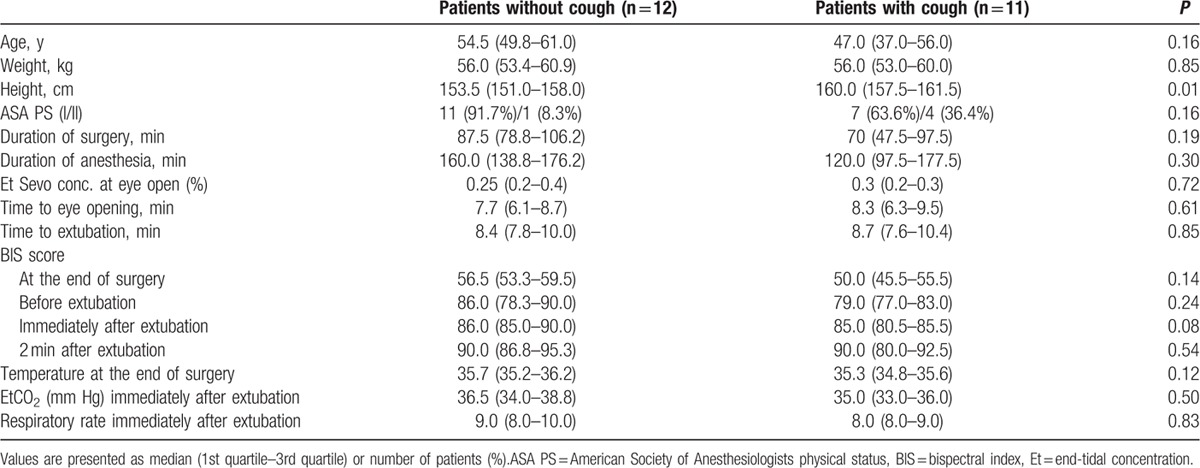
Comparison of recovery profiles during anesthetic emergence between patients without cough and with cough.

**Table 3:**
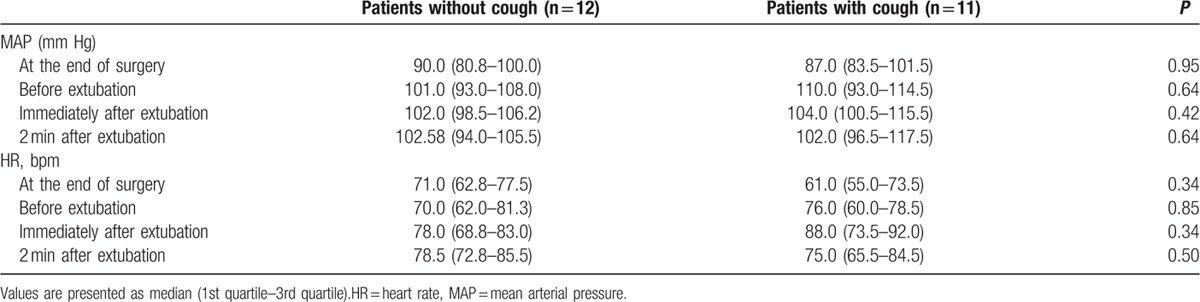
Comparison of hemodynamic profiles during anesthetic emergence between patients without cough and with cough.

No patients suffered any respiratory complications during the emergence period.

## 4 Discussion

The EC_50_ and EC_95_ of remifentanil via effect-site TCI to prevent coughing emergence from sevoflurane anesthesia in female patients requiring DLT intubation was 1.670 and 2.275 ng/mL, respectively. No patient suffered from desaturation, hypoventilation, and all were discharged from PACU without any adverse events.

Several studies have demonstrated the usefulness of opioids for preventing cough. The antitussive effect of opioids is primarily central, and maintaining a certain effect site opioid concentration with TCI is considered reliable.^[7]^ Remifentnil is an ultrashort-acting opioid that is a suitable agent for this purpose, because its effect quickly and predictably disappears after cessation without delayed recovery from anesthesia.^[22,23]^ In several studies, a TCI of remifentanil during anesthetic emergence was reported to allow patients to recover from general anesthesia without coughing or hemodynamic instability,^[8,23,24]^ and several studies extrapolated the effective Ce of remifentanil for preventing airway reflexes during anesthetic emergence.^[7–10]^

Knowledge of the remifentanil concentration in a particular patient undergoing a specific situation (procedure, use of anesthetic agents) is necessary because various factors influence the EC of remifentanil to suppress cough^[9,10,25–27]^: Sex affects the analgesic effects of opioids and the requirements of opioids for preventing cough during extubation is higher in male than in female.^[25,26]^ The type of anesthetic agents used, such as propofol, sevoflurane, and desflurane, change the incidence of coughing during emergence.^[9,27]^ Airway irritation, including that due to an endotracheal tube, also could enhance the cough response.^[10]^

Determining the EC_50_ and EC_95_ of remifentanil for preventing cough in patients undergoing thoracic surgery requiring DLT intubation is important, because the distal tip of the DLT can reach to the main bronchus, which can stimulate different parts of the respiratory tracts compared with SLT. Cough is a defensive reflex of the respiratory tract that can be elicited from the larynx, trachea, carina, or bronchi. These sites are unavoidably stimulated by anesthesiologists during routine practice.^[28]^ The site of stimulation is crucial for determining the patterns of protective response because the protective reflex responses from the respiratory tract are vary, depending on the site of stimulation, with conflicting results.^[12–17]^ In this respect, determination of the EC of remifentanil for the prevention of cough induced by a DLT during anesthetic emergence is required to achieve predictable and smooth emergence, so the investigation of EC_50_ and EC_95_ of remifentanil for these kinds of patients would be clinically significant.^[8]^

In the up-and-down sequential allocation design, there are several methods for estimating EC: the Dixon method, logistic/probit regression, and isotonic regression.^[9]^ Among these methods, we adapted the isotonic regression method for estimating the EC of remifentanil. Because the Dixon method is a simplified design focusing on EC_50_, extrapolation to higher quartiles such the EC_95_ calculated in an up-and-down sequential allocation design may impose great bias and cannot be a reliable value.^[20]^ The estimated EC_95_ of remifentanil to prevent cough cannot be readily applied to clinical practice and should be confirmed in a properly designed study for determining EC_95_, like a biased coin design that can directly estimate EC at any quartile.^[10]^

This study investigated the EC of remifentanil to prevent cough emergence only from sevoflurane anesthesia. Anesthetic agents variously affect the activities of the airway receptors. Volatile anesthetics stimulate the laryngeal receptors^[29]^ and inhibit the pulmonary irritant receptors.^[30]^ Several reports showed that propofol suppresses airway reflexes; propofol decreases the likelihood of laryngospasm and the probability of cough.^[31–34]^ Some studies suggested that emergence cough occurs less frequently and less severely after propofol-based total intravenous anesthesia compared with sevoflurane anesthesia.^[27,31]^ Therefore, knowledge of the EC of remifentanil for preventing cough during emergence for different anesthetic agents, especially propofol, is needed.

This study has several limitations. First, we did not measure real plasma concentrations of remifentanil from patient blood sampling. However, because the Minto pharmacokinetic model, used for remifentanil TCI, has been commonly used with acceptable bias and accuracy in clinical situation,^[35]^ this predicted EC of remifentanil can be used reliably in clinical practice. Second, the study population was limited to females between 20 and 65 years of age. Gender may affect opioid effect; males are less sensitive to the analgesic effects of opioid than females and older patients are more sensitive to opioids.^[8]^ These factors should be considered when interpreting the data.

In conclusion, remifentanil can be a safe and reliable means of cough prevention during emergence from sevoflurane anesthesia after thoracic surgery requiring DLT. The EC_50_ and EC_95_ of remifentanil that suppress coughing is 1.670 and 2.275 ng/mL, respectively.
